# Cell-free DNA methylation reveals cell-specific tissue injury and correlates with disease severity and patient outcomes in COVID-19

**DOI:** 10.1186/s13148-024-01645-7

**Published:** 2024-03-01

**Authors:** Yuan-Yuan Li, Ming-Ming Yuan, Yuan-Yuan Li, Shan Li, Jing-Dong Wang, Yu-Fei Wang, Qian Li, Jun Li, Rong-Rong Chen, Jin-Min Peng, Bin Du

**Affiliations:** 1 Medical ICU, Peking Union Medical College Hospital, Peking Union Medical College and Chinese Academy of Medical Sciences, No.1 Shuaifuyuan, Beijing, 100730 China; 2 Geneplus-Beijing, Floor 9, Building 6, Medical Park Road, Zhongguancun Life Science Park, Changping District, Beijing, 102206 China; 3 Geneplus-Shenzhen, Building B, First Branch, Zhongcheng Life Science Park, Zhongxing Road, Kengzi Street, Pingshan District, Shenzhen, 518000 China

**Keywords:** Circulating-free DNA, Coronavirus disease 2019, Immune response, Tissue of origin inference, Whole genome methylation sequencing

## Abstract

**Background:**

The recently identified methylation patterns specific to cell type allows the tracing of cell death dynamics at the cellular level in health and diseases. This study used COVID-19 as a disease model to investigate the efficacy of cell-specific cell-free DNA (cfDNA) methylation markers in reflecting or predicting disease severity or outcome.

**Methods:**

Whole genome methylation sequencing of cfDNA was performed for 20 healthy individuals, 20 cases with non-hospitalized COVID-19 and 12 cases with severe COVID-19 admitted to intensive care unit (ICU). Differentially methylated regions (DMRs) and gene ontology pathway enrichment analyses were performed to explore the locus-specific methylation difference between cohorts. The proportion of cfDNA derived from lung and immune cells to a given sample (i.e. tissue fraction) at cell-type resolution was estimated using a novel algorithm, which reflects lung injuries and immune response in COVID-19 patients and was further used to evaluate clinical severity and patient outcome.

**Results:**

COVID‑19 patients had globally reduced cfDNA methylation level compared with healthy controls. Compared with non-hospitalized COVID-19 patients, the cfDNA methylation pattern was significantly altered in severe patients with the identification of 11,156 DMRs, which were mainly enriched in pathways related to immune response. Markedly elevated levels of cfDNA derived from lung and more specifically alveolar epithelial cells, bronchial epithelial cells, and lung endothelial cells were observed in COVID-19 patients compared with healthy controls. Compared with non-hospitalized patients or healthy controls, severe COVID-19 had significantly higher cfDNA derived from B cells, T cells and granulocytes and lower cfDNA from natural killer cells. Moreover, cfDNA derived from alveolar epithelial cells had the optimal performance to differentiate COVID-19 with different severities, lung injury levels, SOFA scores and in-hospital deaths, with the area under the receiver operating characteristic curve of 0.958, 0.941, 0.919 and 0.955, respectively.

**Conclusion:**

Severe COVID-19 has a distinct cfDNA methylation signature compared with non-hospitalized COVID-19 and healthy controls. Cell type-specific cfDNA methylation signature enables the tracing of COVID-19 related cell deaths in lung and immune cells at cell-type resolution, which is correlated with clinical severities and outcomes, and has extensive application prospects to evaluate tissue injuries in diseases with multi-organ dysfunction.

**Supplementary Information:**

The online version contains supplementary material available at 10.1186/s13148-024-01645-7.

## Introduction

Circulating cell-free DNA (cfDNA) has emerged as a promising noninvasive biomarker derived from dying cells of various organs. It was widely used to detect fetal chromosomal anomalies, donor-derived DNA for graft rejection and the detection, genotyping, and monitoring of cancer. In these scenarios, genetic differences exist between the DNA nucleotide sequence of the target tissue (fetus, graft, or tumor) and that of the host. However, for diseases affecting a single or multiple organ system within the same genetic background, other information of cfDNA should be integrated.

DNA methylation, as an important epigenetic modification mechanism, plays a vital role in regulating gene expression and maintaining normal physiological functions of organisms. Recently, it was found that each cell type had distinct methylation patterns and it remain conserved and highly stable under physiologic or pathologic conditions [1, 2]. This discovery theoretically allows the tracing of cell-specific DNA methylation patterns to reveal the origins of circulating cfDNA thus shed light on cell death dynamics in health and disease conditions. In this prove-of-concept study, we used COVID-19 as a model to test the hypothesis that cell-specific cfDNA methylation signature could be used to trace disease related cell deaths and it is correlated with disease severities and outcomes.

In Coronavirus disease 2019 (COVID-19), viral infection leads to dysregulation of immune response and uncontrolled inflammation, which are vital triggers of tissue injury [3, 4]. COVID-19 can cause multi-organ failure, but it primarily affects lungs [5, 6]. The prognosis of cases with COVID-19 is related to both inflammatory response and the degree of target organ damage. Newly developed prediction models such as COVID-GRAM and 4C mortality score have showed high discrimination for severity and mortality [7, 8], but these complex scoring systems require a lot of information and calculations. In addition, studies have shown that laboratory markers (e.g. D-dimers, lactate dehydrogenase (LDH), C-reactive protein (CRP) and cytokines like IL-6 and TNF-α) may predict disease severity and prognosis [9, 10]; however, these biomarkers only reflect systemic inflammation levels without specificity. In this case, cfDNA methylation analysis is a good option, which is easy to obtain and can reflect both inflammation and specific tissue injury.

Two previous studies have explored cfDNA methylation signature in COVID-19 related injuries at the organ level [11, 12]. There were controversial findings in regarding the relationship of cfDNA derived from lung with COVID-19 outcome. Recently, Netanel et al. constructed a methylation map of 39 different cell types sorted from 207 healthy human samples and revealed that DNA methylation patterns of the same cell type among individuals are highly conserved. They also identified a large number of cell type-specific methylation markers, which makes it possible to accurately infer the contribution of multiple cell types in a mixture at cell-type resolution [2].

In this study, genome-wide DNA methylation sequencing of cfDNA was performed for COVID-19 patients and healthy persons. The cfDNA methylation profiles were compared between cohorts to investigate the epigenetic features of COVID-19, especially severe patients admitted to intensive care unit (ICU). As this virus primarily affects lungs, and dysregulated immune response is a major contributor of tissue injury, the proportion of cfDNA derived from lung and immune cells to a given sample (i.e. tissue fraction) at cell-type resolution was estimated using a novel algorithm. The proportion of cfDNA derived from different lung cells and immune cells further used to evaluate clinical severity and outcome for COVID-19 patients.

## Methods

### Participants enrollment and sample collection

This study consisted of three cohorts: severe COVID-19, non-hospitalized COVID-19, and healthy control. The severe COVID-19 cohort included severe COVID-19 patients who were admitted to ICU of Peking Union Medical College Hospital (Beijing, China), had positive result of severe acute respiratory syndrome coronavirus 2 (SARS-CoV-2) nucleic acid test and received mechanical ventilation. The healthy cohort and non-hospitalized cohort included healthy volunteers and patients with non-hospitalized COVID-19 from Geneplus-Beijing (Beijing, China) or Geneplus-Shenzhen (Shenzhen, China), respectively. Patients with non-hospitalized COVID-19 had common symptoms on the upper respiratory tract, positive result of SARS-CoV-2 nucleic acid test and no requirement for hospitalization. The diagnosis of COVID-19 was established on the positive result via throat swab testing using the following SARS-CoV-2 nucleic acid detection kits and platforms: DaAn Gene 2019-nCoV detection kit (DaAn Gene, Guangzhou, China), LineGene 9600 Plus Fluorescent Quantitative PCR Instrument (Bioer, Hangzhou, China), MA-6000 Real-time Fluorescent Quantitative PCR Instrument (Molarray, Suzhou, China).

Blood samples were collected within 24 h after ICU admission in severe COVID-19 cohort and were collected one week after diagnosis of COVID-19 in non-hospitalized cohort. For severe COVID-19 patients, demographics, clinical and laboratory information including sequential organ failure assessment (SOFA) score were collected from the electronic medical records on the day of blood collection. Severe lung injury was defined as PaO_2_/FiO_2_ < 100 mmHg, and SOFA score > 6 indicated high SOFA score. All procedures were conducted in accordance with the Declaration of Helsinki. This study was approved by the Ethics Committee of Peking Union Medical College Hospital (Beijing, China) (Approval No.: JS-3480D). Informed consent was obtained from the participants or their representatives.

### DNA extraction, library construction, sequencing and basic bioinformatics analyses

Whole genome methylation sequencing of cfDNA was performed using a TET enzyme-based DNA methylation sequencing platform called GM-seq. The procedures of DNA extraction, library construction and GM-seq were described previously [13]. Blood samples were centrifugated at 1608×*g* for 10 min. The supernatant was then transferred to microcentrifuge tubes and centrifuged at 16,000 × g for another 10 min to remove the remaining cell debris. Plasma cfDNA was extracted from plasma samples using TANBead Maelstrom 2400 extraction instrument (TANBEAD, Taoyuan, China) and MagMAX Cell-Free DNA Isolation Kit (ThermoFisher, Waltham, MA, USA). DNA concentration was measured by Qubit™ dsDNA HS Assay Kit (ThermoFisher, Waltham, MA, USA). The size of cfDNA fragments was assessed using the Qsep100 automated Bio-Fragment Analyzer (Bioptic, New Taipei, China). Before library construction, sequences with CpG totally methylated (positive references) and CpG totally unmethylated (negative references) were mixed into the samples as controls. DNA methylation sequencing libraries were constructed using Hieff NGS® Ultima Pro DNA Library Prep Kit for Illumina (Yeason, Shanghai, China), including end repair, dA tailing, adaptor ligation. Then, 5-methylcytosine (5mC) and 5-hydroxymethylcytosine (5hmC) were oxidized to 5-carboxycytosine (5caC) using the TET2 oxidase, and then converted to dihydrouracil (DHU) under the catalysis of the reducing agent (pyridine borane). DHU can be used as a PCR template and recognized by a DNA polymerase that recognizes U. Through PCR enrichment, 5mC was converted to T for whole genome sequencing, which was performed using Gene + seq2000 sequencer (Geneplus, Suzhou, China). Adaptor sequences and low-quality reads were filtered out from the raw sequencing data using fastp software (v0.19.5) [14]. Clean reads were mapped to the human reference genome (hg19) using Sentieon software (version 202,010). The average sequencing depth in our study was 63 × and the quality control data for cfDNA-based whole genome methylation sequencing was detailed in Additional file 1: Table S1.

### Analysis of differentially methylated regions (DMRs)

The mapping results of samples, positive references and negative references were obtained from BAM files using Samtools (v1.9) [15]. Then, asTair (v3.3.2), a tool that developed for the analysis of bisulfite-free and base-resolution sequencing data generated with modified cytosine to thymine conversion methods, was used to identify methylated sites and evaluate the mean methylation levels of CpG sites. CpG sites with coverage depth ≥ 5× were selected for subsequent differentially methylated regions (DMRs) analysis and principle of principal component analysis (PCA), which were performed using Metilene (v0.2–8) [16] and prcomp function in R package stats (version 4.2.2), respectively. The minimum unit in DMRs analyses was defined as a genomic region comprising at least 10 CpG sites and a distance of no more than 300 bp between adjacent CpGs. DMRs were defined when: (a) the methylation difference level between groups was greater than 0.1 or smaller than − 0.1, and (b) the *q*-value was smaller than 0.1. DMRs were then annotated to different functional regions, including promoters, introns, exons and intergenic regions, using R package genomation (version 3.17). Venn diagram was plotted using an online tool named Venny (2.1). DMRs were used to perform gene ontology (GO) pathway enrichment analyses using R package clusterProfiler (version 4.7.1.003). Unsupervised clustering was performed using R package pheatmap (version 1.0.12).

### Origin inference of tissue injury

As DNA methylation patterns of the same cell type among individuals are highly conserved, the methylation data for main human cell types were obtained from previous literatures to screen cell type-specific methylation markers (genomic regions containing at least 3 CpG sites) [2, 17] The specificity of any genomic region containing at least 3 CpGs was calculated for all cell types, which was defined as the proportion of reads with totally unmethylated CpGs at this genomic region in the corresponding cells. A marker was selected if all of the following criteria were met: (1) the methylation pattern of this genomic region was highly conserved among individuals; (2) the difference of specificity between target cell type and all background cell types was greater than 0.2; (3) the specificity of background cell types was smaller than 0.25. As a result, a total of 1010, 406, 24, 23, 22, 21, 23 and 33 markers were screened out for lung cells (alveolar epithelial cells, bronchial epithelial cells), immune cells (natural killer cells, granulocytes, monocytes and macrophages, B cells, T cells) and lung endothelial cells.

To infer the relative proportion of DNA released by target cells in cfDNA, tissue fraction was used to reflect the positive signals derived from target cells in a given sample. The methylation state of sequencing reads for a given marker was annotated as U (≤ 25% methylated CpGs), M (≥ 75% methylated CpGs) or X (25% < methylated CpGs < 75%) [2]. Raw tissue fraction for a specific cell type was calculated as the ratio of the sum of U reads to the sum of all reads for all markers. In order to further reduce background noise and improve robustness of the algorithm, raw tissue fraction for human cell types was calculated in 30 healthy persons, and at most 20 cell types with consistently low tissue fraction among individuals, representing that their cfDNA fragments were rarely released into the blood under normal physiologic condition, were selected as reference. Tissue fraction for these cell types were considered as the level of background noise. For a given sample, the mean tissue fraction of reference cell types whose tissue fraction did not exceed the background noise threshold (95% quantile) was used as the individualized adjustment coefficient. The adjusted tissue fraction for a specific target cell type was calculated as: raw tissue fraction × (1–adjustment coefficient). R package pROC (version 1.18.0) was used to draw receiver operating characteristic (ROC) curves.

### Statistical analyses

Data are presented as the frequency and percentage for categorical variables and means ± standard deviation (SD) or median with interquartile range (IQR) for continuous variables, depending on the nature and distribution of variables. The normality of data was tested using the Shapiro–Wilk normality test. Group comparisons of continuous parameters were performed using Student’s t test or Mann–Whitney nonparametric test as appropriate. All functions used belongs to R package stats (version 4.2.2). Two-tailed *P* < 0.05 was considered statistically significant.

## Results

### Global cfDNA methylation is reduced in COVID‑19 patients

**Fig. 1:**
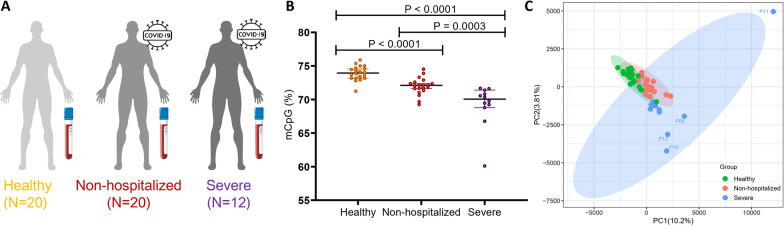
Comparison of methylation levels among three cohorts. **A** Overview of three cohorts for DNA methylation profiles comparison. **B** The percentage of methylated CpG sites in three cohorts; **C** principal component analysis of CpG methylation levels

This study included 20 cases with non-hospitalized COVID-19, 12 cases with severe COVID-19 admitted to ICU, and 20 healthy individuals as control group (Fig. 1A). Patients with severe COVID-19 were significant older than those in non-hospitalized cohort (63 ± 14 years vs 31 ± 6 years, *P* < 0.001). Male accounted for 66.7% and 35.0% of cases in severe and non-hospitalized cohort, respectively (*P* = 0.080). Median age of the healthy cohort was 51 (IQR, 40–55) years, and 50% of the patients were male. Clinical data in severe cohort are shown in Table 1. Time from symptom to blood collection were 18 ± 9 days in severe cohort. 33.3% of patients with severe COVID had SOFA score more than 6 on ICU admission. All of them received invasive mechanical ventilation, and 4 (33.3%) received extracorporeal membrane oxygenation (ECMO) support. All-cause in-hospital mortality occurred in 4/12 (33.3%) and the length of ICU stay was 19 (IQR, 7–30) days.

**Table 1:** Characteristics of COVID-19 patients admitted to ICU

Variables	Patients admitted to ICU (*n* = 12)
Male	8 (66.7)
Age (years)	63 ± 14
Days from symptom to blood collection	18 ± 9
*Laboratory results*	
White blood cells (× 10^9^/L)	9.62 ± 3.51
Neutrophils (× 10^9^/L)	7.75 ± 4.21
Lymphocytes (× 10^9^/L)	0.64 (0.32, 0.97)
Monocytes (× 10^9^/L)	0.21 ± 0.09
D-dimer (mg/L)	2.65 (1.47, 4.27)
*Disease severity*	
APACHE II score	17 ± 5
SOFA score	7 ± 2
SOFA score > 6	5 (41.7)
PaO_2_/FiO_2_ (mmHg)	123.58 ± 56.14
PaO_2_/FiO_2_ < 100 mmHg	5 (41.7)
IMV	12 (100.0)
ECMO	4 (33.3)
Septic shock	3 (25.0)
*Outcome*	
In-hospital death	4 (33.3)
Length of ICU stay (days)	19 (7, 30)

Data are present as *n* (%), mean ± standard deviation or median (interquartile range) as appropriate

*APACHE* Acute Physiology and Chronic Health Evaluation; *COVID-19* Coronavirus disease 2019; *ECMO* Extracorporeal membrane oxygenation; *ICU* Intensive care unit; *IMV* Invasive mechanical ventilation; *SOFA* Sequential organ failure assessment

The median global methylation level in healthy, non-hospitalized and severe cohort was 73.95%, 72.11% and 70.06%, respectively. Severe cohort had the lowest global methylation level compared with healthy and non-hospitalized cohorts (*P* < 0.0001 and *P* = 0.0003). In addition, the global methylation burden of non-hospitalized COVID-19 was significantly lower than healthy persons (*P* < 0.0001) (Fig. 1B). Subsequently, we performed PCA on CpG sites to examine the global methylation differences among three cohorts (Fig. 1C). It revealed that the methylation patterns of healthy individuals and non-hospitalized patients were similar, while the methylation pattern of severe COVID-19 was distinct from other cohorts. Among severe COVID-19 patients, the methylation patterns of 4 cases (P06, P10, P11 and P12) were far away from others, suggesting the heterogeneity of severe COVID-19. Interestingly, all the 4 scattered cases received ECMO support while others did not.

### Severe COVID-19 patients had distinct cfDNA methylation profiles

**Fig. 2:**
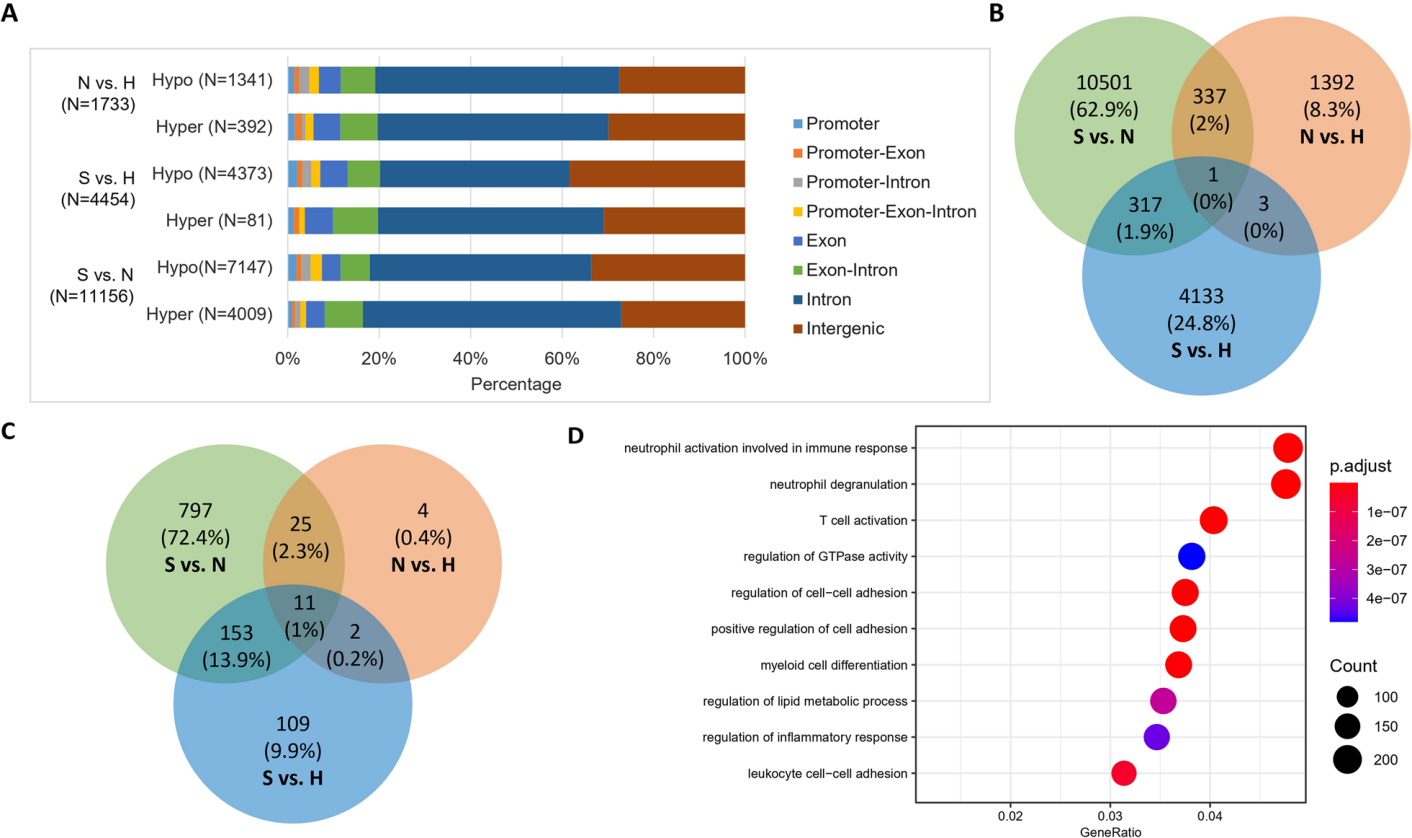
Analysis of differentially methylated regions (DMRs) for three cohorts. **A** The distributions of DMRs in different genomic regions; **B** Venn diagram with the number of shared DMRs across different cohorts; **C** Venn diagram showing the number of shared pathways across different cohorts obtained from GO pathway enrichment analysis of DMRs. **D** Dotplot showing the top ten gene ontological (GO) biological processes related to the DMRs between non-hospitalized and severe COVID-19 cohorts. H, healthy; N, non-hospitalized; S, severe

To further investigate the methylation levels on specific locus related to severe COVID-19, we performed pairwise comparison of three cohorts to find DMRs. A total of 1733, 4454 and 11,156 DMRs were identified for the comparison of non-hospitalized—healthy, severe—healthy and severe—non-hospitalized, respectively, suggesting the large methylation difference between severe and non-hospitalized COVID-19. Compared with healthy persons, 77.4% and 98.2% of DMRs were hypo-methylated regions in patients with non-hospitalized and severe COVID-19, respectively. Compared with non-hospitalized cohort, 64.1% of DMRs were hypo-methylated regions in severe COVID-19. A majority of DMRs reside within intron and intergenic regions and the proportion of DMRs in promoter regions was 6.5%, 7.0% and 7.2% for the comparison of non-hospitalized—healthy, severe—healthy and severe—non-hospitalized, respectively (Fig. 2A). In addition, the number of hypo-methylated DMRs in promoter regions was more than hyper-methylated DMRs in terms of the comparison of severe—non-hospitalized cohorts (hypo-methylated: 7.5% (533/7147)—hyper-methylated: 4.0% (161/4009), *P* < 0.0001). We observed the sharing of DMRs between severe—non-hospitalized and severe—healthy, severe—non-hospitalized and non-hospitalized—healthy, while severe—healthy shared less DMRs with non-hospitalized—healthy (Fig. 2B).

GO pathway enrichment analysis was performed to further examine the cellular function of DMRs (Fig. 2D and Additional file 2: Fig. 1A and 1B). A total of 42, 275 and 286 pathways were enriched for the comparison of non-hospitalized—healthy, severe—healthy and severe—non-hospitalized, respectively. Compared with sharing DMRs analysis, the proportions of shared pathways were higher (Fig. 2C). The top ten pathways related to the DMRs between non-hospitalized and severe COVID-19 cohorts are illustrated in Fig. 2D. Most of them are involved in immune response, including neutrophil activation, neutrophil degranulation, T cell activation, myeloid cell differentiation and leukocyte cell–cell adhesion. The methylation levels of DMRs for non-hospitalized and severe COVID-19 patients were used for unsupervised clustering, which revealed that DNA methylation level of DMRs was correlated with the severity of COVID-19. Based on methylation pattern of DMRs, COVID-19 patients could be classified into two groups: one group included 11 severe and 3 non-hospitalized COVID-19 patients and another group included 1 severe and 17 non-hospitalized COVID 19 cases (Additional file 2: Fig. 1C), suggesting the distinct methylation difference between severe and non-hospitalized COVID-19.

### Lung injury in COVID-19 patients traced by cfDNA methylation

**Fig. 3:**
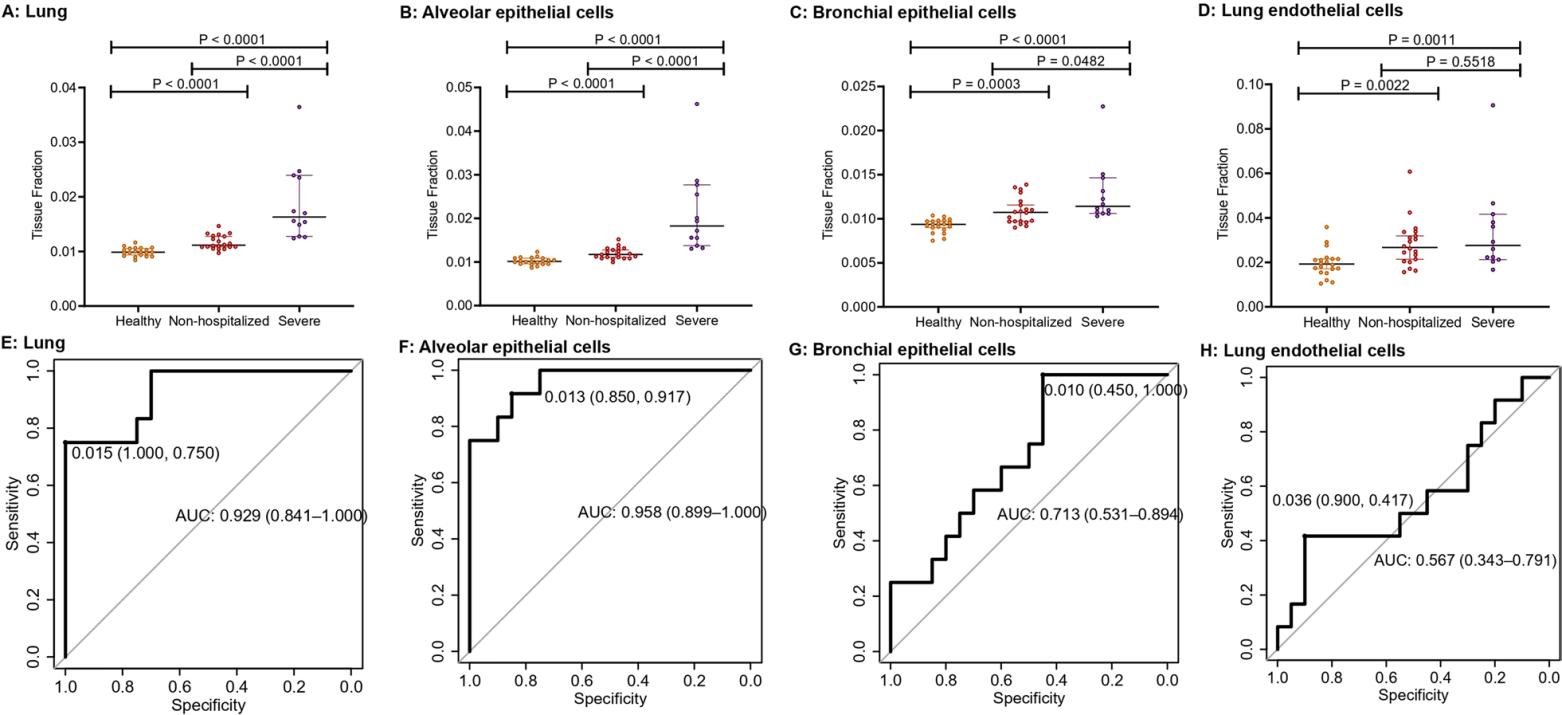
Comparison of cfDNA derived from different pulmonary cell types among three cohorts (**A**: lung; **B**: alveolar epithelial cells; **C**: bronchial epithelial cells; **D**: lung endothelial cells) and the receiver operating characteristic curves using tissue fractions to classify severe and non-hospitalized COVID-19 patients (**E**: lung tissue; **F**: alveolar epithelial cells; **G**: bronchial epithelial cells; **H**: lung endothelial cells)

We next assessed the presence of lung-derived cfDNA in the plasma of patients with COVID-19. Severe cohort had a significantly higher level of cfDNA from lung (Fig. 3A; *P* < 0.0001, *P* < 0.0001), lung alveolar epithelial cells (Fig. 3B; *P* < 0.0001, *P* < 0.0001) and bronchial epithelial cells (Fig. 3C; *P* = 0.0482, *P* < 0.0001) than non-hospitalized and healthy cohorts. Non-hospitalized cohort also had a significantly higher level of cfDNA for lung (Fig. 3A; *P* < 0.0001), lung alveolar epithelial cells (Fig. 3B; *P* < 0.0001) and bronchial epithelial cells (Fig. 3C; *P* = 0.0003) than healthy individuals. Plasma cfDNA derived from for lung endothelial cells in severe and non-hospitalized cohorts was significantly higher than that in healthy cohort (*P* = 0.0011 and 0.0022), while no significant difference was observed between severe and non-hospitalized cases (Fig. 3D; *P* = 0.5518). Plasma cfDNA derived from lung (Fig. 3E), alveolar epithelial cells (Fig. 3F), bronchial epithelial cells (Fig. 3G) and lung endothelial cells (Fig. 3H) was then used to classify severe and non-hospitalized cases, with the area under the curve (AUC) of ROC of 0.929 (95 confidence interval (CI) 0.841–1.000), 0.958 (95% CI 0.899–1.000), 0.713 (95% CI 0.531–0.894) and 0.567 (95% CI 0.343–0.791), respectively. Plasma cfDNA derived from alveolar epithelial cells had the optimal performance to discriminate severe COVID-19 from non-hospitalized cases.

### Abnormal presence of immune cells in COVID-19 patients traced by cfDNA methylation

**Fig. 4:**
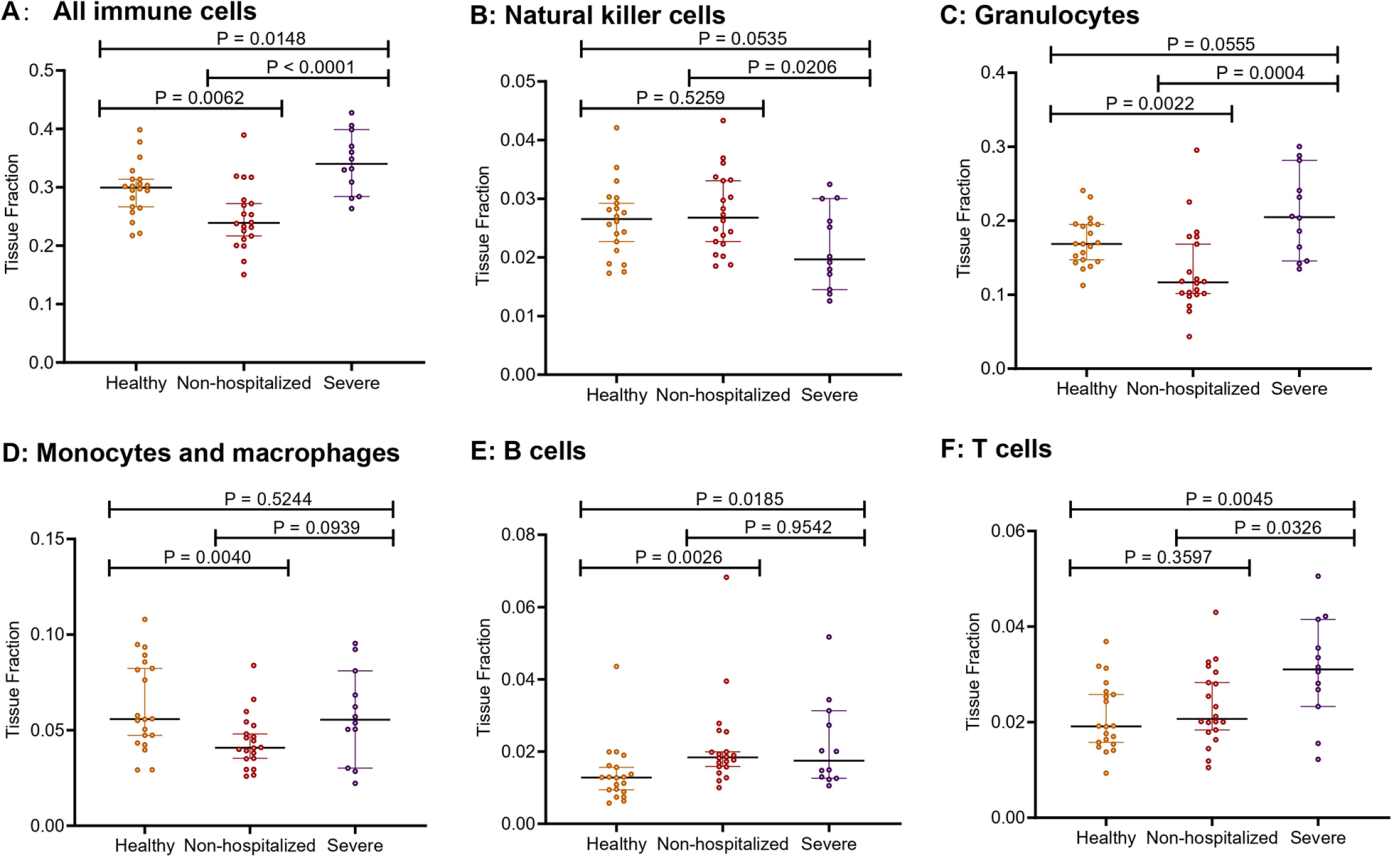
Comparison of cfDNA derived from for different immune cell types among three cohorts (**A**: all immune cells; **B**: natural killer cells; **C**: granulocytes; **D**: monocytes and macrophages; **E**: B cells; **F**: T cells)

The proportion of cfDNA derived from immune cells was then compared between cohorts. As shown in Fig. 4A, cfDNA from all immune cells was significantly higher in severe cohort compared with healthy (*P* = 0.0148) and non-hospitalized (*P* < 0.0001) cohorts, and non-hospitalized COVID-19 patients had significantly lower cfDNA than healthy individuals (*P* = 0.0062). Specifically, severe COVID-19 had significantly lower cfDNA from for natural killer cells than non-hospitalized cohort (*P* = 0.0206) (Fig. 4B), higher cfDNA from granulocytes compared with non-hospitalized cohort (*P* = 0.0004) (Fig. 4C), higher cfDNA from B cells compared with healthy persons (*P* = 0.0185) (Fig. 4E) and higher cfDNA from T cells compared with non-hospitalized (*P* = 0.0326) and healthy (*P* = 0.0045) cohorts (Fig. 4F). Compared with healthy controls, non-hospitalized COVID-19 had elevated cfDNA from B cells (*P* = 0.0026) (Fig. 4 E) and reduced cfDNA from granulocytes (*P* = 0.0022) (Fig. 4C) and monocytes (*P* = 0.0040) (Fig. 4D).

### Damage of alveolar epithelial cells was correlated with severity and outcome of COVID-19

**Fig. 5:**
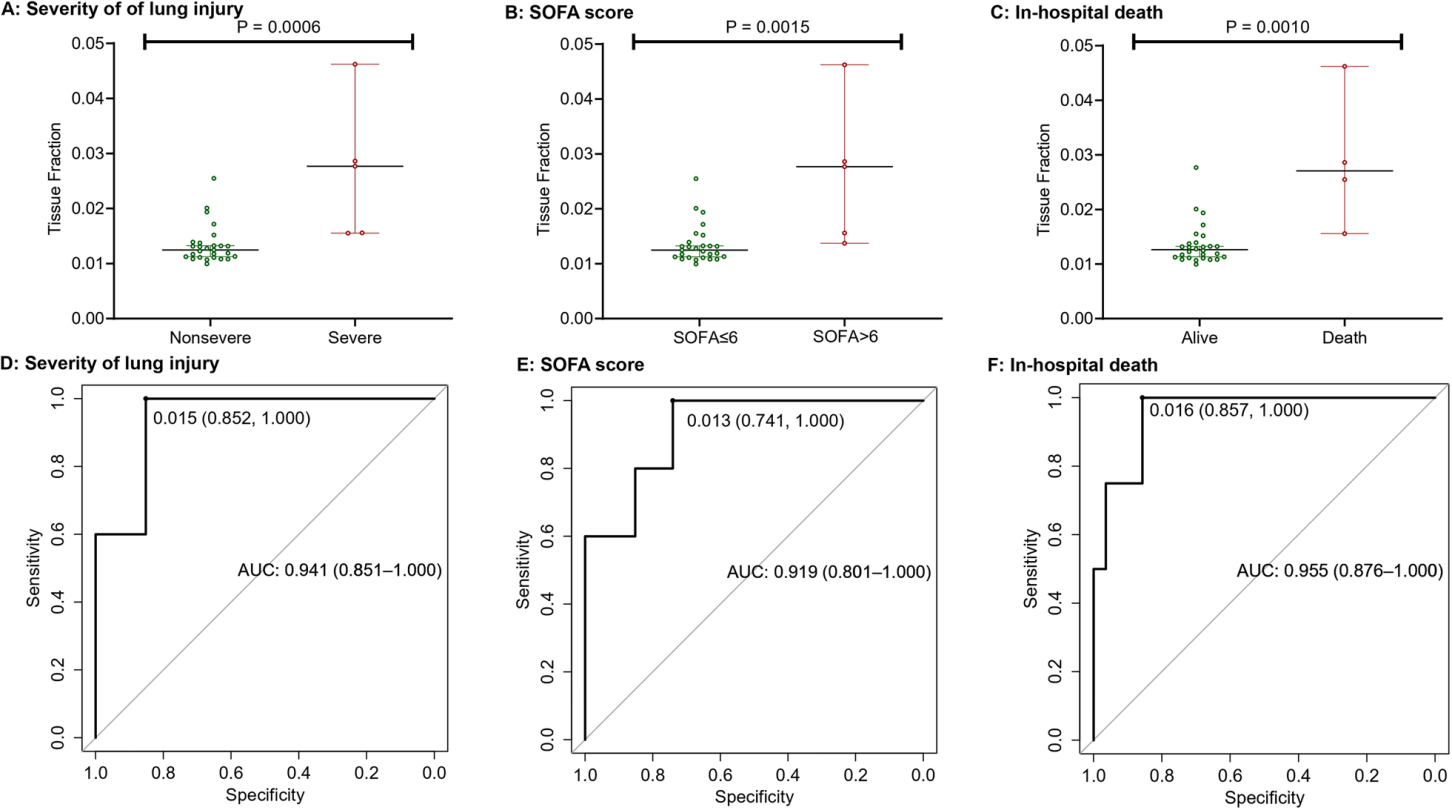
Comparison of cfDNA derived from alveolar epithelial cells among COVID-19 patients with different disease severities and outcomes, and the receiver operating characteristic curves using tissue fractions to classify severities and outcomes: **A** and **D** severity of lung injury; **B** and **E** sequential organ failure assessment (SOFA) score; **C** and **F** in-hospital death

Given the optimal performance of cfDNA derived from alveolar epithelial cells to discriminate severe COVID-19 from non-hospitalized cases, we further examined its relationship with severity of lung injury, SOFA score > 6 and in-hospital death in COVID-19 patients. Patients with severe lung injury has significantly higher tissue fraction than patients without severe lung injury (*P* = 0.0006) (Fig. 5A). Higher tissue fraction was also observed for patients with SOFA score > 6 (Fig. 5B) and in-hospital death (Fig. 5C). Plasma cfDNA derived from alveolar epithelial cells was then used to classify severity of lung injury, SOFA score and predict mortality. The AUC was 0.941 (95% CI 0.851–1.000), 0.919 (95% CI 0.801–1.000) and 0.955 (95% CI 0.876–1.000), respectively (Fig. 5D–F).

## Discussion

Previously, most peripheral blood-based DNA methylation studies used genomic DNA isolated from whole blood or peripheral blood mononuclear cells (PBMC) to investigate the methylation signatures of COVID-19 and their relationship with clinical severities and outcomes [18–22], while the significance of cfDNA methylation in COVID-19 progression has not been fully understand [11, 12, 23]. This study comprehensively described the distinct cfDNA methylation features related to COVID-19, and evaluated cell death events related to lung and immune cells in COVID-19 through genome-wide DNA methylation sequencing of COVID-19 patients and healthy controls. The elevated cfDNA derived from for alveolar epithelial cells was correlated with clinical severities and outcomes in patients with COVID-19.

Our study observed that the global cfDNA methylation levels decreased with the increasing of disease severity, and the methylation pattern of severe patients obviously differed from non-hospitalized and healthy cases. These findings suggested that patients with severe disease had more abnormalities of methylation and more uncontrolled gene expression. DMR analyses among three groups showed that there were more hypo-methylated DMRs in the two COVID-19 groups, and there were significantly higher number of hypo-methylated DMRs than hyper-methylated DMRs in the promoter region for the comparison of severe and non-hospitalized cases, indicating that more genes in the case group were hypo-methylated, leading to abnormal activation of gene expression. These findings were consistent with the previous work conducted by Balnis et al., revealing higher numbers of hypo-methylated DMRs than hyper-methylated DMRs by comparing the cfDNA methylation profiles of 15 patients hospitalized with COVID-19 and 15 healthy volunteers [23].

In addition, we found that severe COVID-19 had distinct cfDNA methylation profiles compared with non-hospitalized cohort with the identification of 11,156 DMRs and enrichment of 286 pathways. Most of the top ten pathways were involved in immune response. It is reasonable in terms of the defense between immune system and COVID-19. Patients with severe COVID-19 experience the coexistence of immunosuppression and hyperinflammation states, characterized by the decrease of lymphocytes and elevation of inflammatory cytokines, respectively [24]. Compared with non-severe cases, severe cases tend to have lower lymphocytes and higher leukocytes, infection-related biomarkers and inflammatory cytokines [4]. The DMRs between severe and non-hospitalized cases were enriched in neutrophil activation and degranulation, T cell activation and leukocytes cell–cell adhesion, inflammatory response regulation and myeloid cell differentiation pathways, which was consistent with the difference of immune response between severe and non-severe patients [4, 24, 25]. Multiple studies confirmed the connection between GTPase signaling regulation and acute respiratory distress syndrome (ARDS) and subsequent pulmonary fibrosis [26], which was consistent with our GO pathway enrichment findings.

The tissue fraction for immune cells obtained by cfDNA methylation sequencing reflects cell death events of immune cells in vivo rather than the number of immune cells in blood [27]. The death of immune cells may result from the drastic immune response that they participate in, or lymphopenia caused by virus infection. In the early stage of COVID-19 infection (usually within a few hours as other virus’ infection), tissue-resident innate immune cells, particularly neutrophils and monocytes in the nasopharyngeal mucosa, are stimulated by chemokines. They initially recognize the viral infection and recruit more innate immune cells to eliminate the virus. Adaptive immunity, including B cells and T cells, starts participating in the antiviral process a few days after infection [28]. Most patients (including asymptomatic and mild COVID-19 patients) can clear the virus, and the counts of lymphocytes as well as immune response will gradually return to normal levels. While severe cases experience a hyperinflammation phase, accompanied by persistently lower levels of lymphocytes called lymphopenia [24]. In our study, plasma samples were collected 7 days post-infection in the non-hospitalized patients. Adaptive immunity is likely playing a dominant role at this time point. Thus, it was reasonable that active adaptive immune response may result in more cell death of B cells and thus elevated cfDNA methylation markers of B cells. In contrast, severe COVID-19 patients who had blood collected immediately after ICU admission were undergoing a phase of hyperinflammation and lymphopenia. Therefore, the cfDNA methylation markers of B cells, T cells, and granulocytes were all elevated due to increased cell death of these cells. In addition, previous studies observed the accumulation of more natural killer cells in mild COVID-19 rather than severe COVID-19, suggesting the potential role of natural killer cells to prevent over-inflammation and tissue injury [29–33], which may explain the reduced cfDNA from natural killer cells in severe COVID-19 compared with non-hospitalized patients. Plasma cfDNA derived from immune cells provides a novel biomarker to for monitoring immune responses in patients with COVID-19.

A panel of lung-specific methylation markers, targeting alveolar and bronchial epithelial cells of lung was applied to access lung-derived cfDNA in plasma samples from healthy individuals, patients with lung cancer, and patients with chronic obstructive pulmonary disease. The study revealed that normal lung cell turnover likely releases cfDNA into the air spaces, rather than to the bloodstream [34]. Lung-derived cfDNA is observed in the plasma when there is a pathological disruption of lung tissue architecture, as seen in lung cancer and to a lesser extent in other lung diseases [34]. Significant lung injury was observed in COVID-19 patients in our study, indicating that lung damage in COVID-19 is sufficient to reverse tissue topology and release cfDNA to blood rather than to the air spaces.

We also found that as the disease worsened, the lung epithelial cells were more severely damaged, especially for the alveolar epithelial cells. Tissue fraction for alveolar epithelial cells had the best performance to distinguish severe cases from non-hospitalized cases. These findings were in accordance with the respiratory epithelial cell responses to SARS-CoV-2. In conducting airways, ciliated cells and secretory cells are the main cells infected and their response to interferon and cytokine is moderate after SARS-CoV-2 infection. The alveolar epithelial cell is the main target cell type in the gas exchange portion of the lung. Alveolar cell death and marked innate immune response during infection likely impede epithelial repair mechanisms and contribute to alveolar damage and resultant acute respiratory distress syndrome [35].

Two previous studies [11, 12], respectively, conducted whole genome bisulfite sequencing of plasma cfDNA from COVID-19 patients. Based on the publicly available reference methylation atlas of human tissues, they deconvoluted the tissue origins using CelFiE algorithm and a non-negative least-squares method (not specified), respectively. Then, they multiplied the relative estimated proportions of cfDNA for lung by the total concentration of cfDNA in plasma to ascertain the absolute cfDNA concentration of lung. In Andargie et al.’s study [11], lung cfDNA level was correlated with COVID-19 disease severity and outcome, showing an AUC of 0.938 and 0.847, respectively. It is noteworthy that samples from Cheng et.al.'s research were sequenced to a minimum depth of 0.7 × human genome coverage. We speculated that this low sequencing depth might lead to inaccurate identification of low-abundance signals in cfDNA, and thus the AUC of 0.56 for lung cfDNA in predicting WHO ordinal scores [12]. The average sequencing depth in our study was 63x, and our study utilized cell type-specific methylation markers from Netanel et.al.'s research to pinpoint lung damage to alveolar epithelial cells [2], which could effectively differentiate COVID-19 cases with varying severities, lung injury levels, SOFA scores, and in-hospital deaths, achieving AUCs of 0.958, 0.941, 0.919, and 0.955, respectively. Therefore, our biomarker offers a more accurate insight into tissue injury and outperforms previous studies in its performance.

Several limitations needed to be disclosed in the study. Firstly, this study included a limited number of samples, especially for patients with severe COVID-19. The findings of this study need to be further validated in a large cohort study. In addition, all patients that transferred to our department already progressed to severe disease, and samples from moderate COVID-19 patients were not included in this study. Furthermore, the blood for severe and non-hospitalized patients was collected 17 days after symptom presentation and 7 days after diagnosis, respectively. It is more interesting to further explore the specific cfDNA methylation profile for COVID-19 patients at the initial stage of infection, and the performance of cfDNA from lung alveolar epithelial cell to predict COVID-19 severity, prognosis and death. Moreover, few patients in our study developed multi-organ dysfunction, which makes it impossible to investigate the relationship between cfDNA methylation signatures and injuries derived from other tissue or cell types. Plasma cfDNA methylation signatures may have potential applications in other settings, warranting future investigation.

## Conclusion

In conclusion, this study suggested that severe COVID-19 had a distinct cfDNA methylation signature compared with non-hospitalized COVID-19 and healthy controls. Furthermore, a novel method was developed to infer origin of tissue injury at cell-type resolution, and significant lung injury was observed in COVID-19 patients, especially injury derived from alveolar epithelial cells. Plasma cfDNA derived from alveolar epithelial cells had the optimal performance to differentiate COVID-19 with different severities and outcomes. This study demonstrated the potential of cfDNA in tracing COVID-19 related tissue damage and indicating disease severity and clinical outcome, which has extensive application prospects to evaluate tissue injuries in diseases with multi-organ dysfunction.

### Supplementary Information

**Additional file 1: Table S1.** The detailed quality control data for cfDNA-based whole genome methylation sequencing.

**Additional file 2: Fig. 1.** Dotplots showing the top ten gene ontological (GO) biological processes related to the DMRs between **A** severe and healthy cohorts and **B** non-hospitalized and healthy cohorts; **C** heatmap of unsupervised clustering using differentially methylated regions between non-hospitalized and severe COVID-19 patients.

## Data Availability

The datasets generated and/or analyzed during the current study are available in the Genome Sequence Archive (GSA) repository, [ACCESSION NUMBER: HRA006758].

## Abbreviations

APACHE: Acute Physiology and Chronic Health Evaluation
AUC: Area under the curve
cfDNA: Circulating-free DNA
CI: Confidence interval
COVID-19: Coronavirus disease 2019
CRP: C-reactive protein
DHU: Dihydrouracil
DMRs: Differentially methylated regions
ECMO: Extracorporeal membrane oxygenation
GO: Gene ontology
ICU: Intensive care unit
LDH: Lactate dehydrogenase
PCA: Principal component analysis
ROC: Receiver operating characteristic
SARS-CoV-2: Severe acute respiratory syndrome coronavirus 2
SOFA: Sequential organ failure assessment
PBMC: Peripheral blood mononuclear cells
ARDS: Acute respiratory distress syndrome

## References

[1] Bergman Y, Cedar H (2013). DNA methylation dynamics in health and disease. Nat Struct Mol Biol.

[2] Loyfer N, Magenheim J, Peretz A, Cann G, Bredno J, Klochendler A (2023). A DNA methylation atlas of normal human cell types. Nature.

[3] Tay MZ, Poh CM, Rénia L, MacAry PA, Ng LFP (2020). The trinity of COVID-19: immunity, inflammation and intervention. Nat Rev Immunol.

[4] Qin C, Zhou L, Hu Z, Zhang S, Yang S, Tao Y (2020). Dysregulation of immune response in patients with coronavirus 2019 (COVID-19) in Wuhan, China. Clin Infect Dis.

[5] Guan WJ, Ni ZY, Hu Y, Liang WH, Ou CQ, He JX (2020). China medical treatment expert group for Covid-19 clinical characteristics of coronavirus disease 2019 in China. N Engl J Med.

[6] Huang C, Wang Y, Li X, Ren L, Zhao J, Hu Y (2020). Clinical features of patients infected with 2019 novel coronavirus in Wuhan, China. Lancet.

[7] Liang W, Liang H, Ou L, Chen B, Chen A, Li C, et al; China Medical Treatment Expert Group for COVID-19. Development and validation of a clinical risk score to predict the occurrence of critical illness in hospitalized patients with COVID-19. JAMA Intern Med. 2020;180(8):1081–1089. 10.1001/jamainternmed.2020.2033 10.1001/jamainternmed.2020.2033 PMC7218676 32396163

[8] Knight SR, Ho A, Pius R, Buchan I, Carson G, Drake TM (2020). Risk stratification of patients admitted to hospital with covid-19 using the ISARIC WHO clinical characterisation protocol: development and validation of the 4C mortality score. BMJ.

[9] Gallo Marin B, Aghagoli G, Lavine K, Yang L, Siff EJ, Chiang SS (2021). Predictors of COVID-19 severity: a literature review. Rev Med Virol.

[10] Luporini RL, Rodolpho JMA, Kubota LT, Martin ACBM, Cominetti MR, Anibal FF, Pott-Junior H (2021). IL-6 and IL-10 are associated with disease severity and higher comorbidity in adults with COVID-19. Cytokine.

[11] Andargie TE, Tsuji N, Seifuddin F, Jang MK, Yuen PS, Kong H (2021). Cell-free DNA maps COVID-19 tissue injury and risk of death and can cause tissue injury. JCI Insight.

[12] Cheng AP, Cheng MP, Gu W, Sesing Lenz J, Hsu E, Schurr E (2021). Cell-free DNA tissues of origin by methylation profiling reveals significant cell, tissue, and organ-specific injury related to COVID-19 severity. Med.

[13] Chen X, Liu J, Li J, Xie Y, Yu Z, Shen L, Liu Q, Wu W, Zhao Q, Lin H, Liu G, Luo Q, Yang L, Huang Y, Zhao M, Yi X, Xia X (2023). Identification of DNA methylation and genetic alteration simultaneously from a single blood biopsy. Genes Genom.

[14] Chen S, Zhou Y, Chen Y, Gu J (2018). fastp: an ultra-fast all-in-one FASTQ preprocessor. Bioinformatics.

[15] Danecek P, Bonfield JK, Liddle J, Marshall J, Ohan V, Pollard MO, Whitwham A, Keane T, McCarthy SA, Davies RM, Li H (2021). Twelve years of SAMtools and BCFtools. Gigascience.

[16] Jühling F, Kretzmer H, Bernhart SH, Otto C, Stadler PF, Hoffmann S (2016). metilene: fast and sensitive calling of differentially methylated regions from bisulfite sequencing data. Genome Res.

[17] Magenheim J, Rokach A, Peretz A, Loyfer N, Cann G, Amini H (2022). Universal lung epithelium DNA methylation markers for detection of lung damage in liquid biopsies. Eur Respir J.

[18] Wang G, Xiong Z, Yang F, Zheng X, Zong W, Li R, Bao Y (2022). Identification of COVID-19-associated DNA methylation variations by integrating methylation array and scRNA-Seq data at cell-type resolution. Genes.

[19] Bowler S, Papoutsoglou G, Karanikas A, Tsamardinos I, Corley MJ, Ndhlovu LC (2022). A machine learning approach utilizing DNA methylation as an accurate classifier of COVID-19 disease severity. Sci Rep.

[20] Balnis J, Madrid A, Hogan KJ, Drake LA, Chieng HC, Tiwari A (2021). Blood DNA methylation and COVID-19 outcomes. Clin Epigenetics.

[21] Barturen G, Carnero-Montoro E, Martínez-Bueno M, Rojo-Rello S, Sobrino B, Porras-Perales Ó (2022). Whole blood DNA methylation analysis reveals respiratory environmental traits involved in COVID-19 severity following SARS-CoV-2 infection. Nat Commun.

[22] Corley MJ, Pang APS, Dody K, Mudd PA, Patterson BK, Seethamraju H (2021). Genome-wide DNA methylation profiling of peripheral blood reveals an epigenetic signature associated with severe COVID-19. J Leukoc Biol.

[23] Balnis J, Madrid A, Hogan KJ, Drake LA, Adhikari A, Vancavage R (2023). Whole-genome methylation sequencing reveals that COVID-19-induced epigenetic dysregulation remains 1 year after hospital discharge. Am J Respir Cell Mol Biol.

[24] Liu Y, Li Y, Xu D, Zhang J, Peng Z (2021). Severe COVID-19: Immunosuppression or hyperinflammation?. Shock.

[25] Qin G, Liu S, Yang L, Yu W, Zhang Y (2021). Myeloid cells in COVID-19 microenvironment. Signal Transduct Target Ther.

[26] Pollard CA, Morran MP, Nestor-Kalinoski AL (2020). The COVID-19 pandemic: a global health crisis. Physiol Genom.

[27] Fox-Fisher I, Piyanzin S, Ochana BL, Klochendler A, Magenheim J, Peretz A (2021). Remote immune processes revealed by immune-derived circulating cell-free DNA. Elife.

[28] Schultze JL, Aschenbrenner AC (2021). COVID-19 and the human innate immune system. Cell.

[29] Deng X, Terunuma H, Nieda M (2022). Exploring the utility of NK cells in COVID-19. Biomedicines.

[30] Chen H, Liu W, Wang Y, Liu D, Zhao L, Yu J (2021). SARS-CoV-2 activates lung epithelial cell proinflammatory signaling and leads to immune dysregulation in COVID-19 patients. EBioMedicine.

[31] Liao M, Liu Y, Yuan J, Wen Y, Xu G, Zhao J (2020). Single-cell landscape of bronchoalveolar immune cells in patients with COVID-19. Nat Med.

[32] Shaath H, Vishnubalaji R, Elkord E, Alajez NM (2020). Single-cell transcriptome analysis highlights a role for neutrophils and inflammatory macrophages in the pathogenesis of severe COVID-19. Cells.

[33] Ren X, Wen W, Fan X, Hou W, Su B, Cai P (2021). COVID-19 immune features revealed by a large-scale single-cell transcriptome atlas. Cell.

[34] Zhou Y, He Y, Yang H, Yu H, Wang T, Chen Z (2020). Development and validation a nomogram for predicting the risk of severe COVID-19: a multi-center study in Sichuan, China. PLoS ONE.

[35] Bridges JP, Vladar EK, Huang H, Mason RJ (2022). Respiratory epithelial cell responses to SARS-CoV-2 in COVID-19. Thorax.

