# Supplementary figures and images for: Cell-free DNA methylation reveals cell-specific tissue injury and correlates with disease severity and patient outcomes in COVID-19
Source: Clin Epigenetics. 2024 Mar 1;16:37. doi: 10.1186/s13148-024-01645-7 (PMC10908074; doi:10.1186/s13148-024-01645-7)

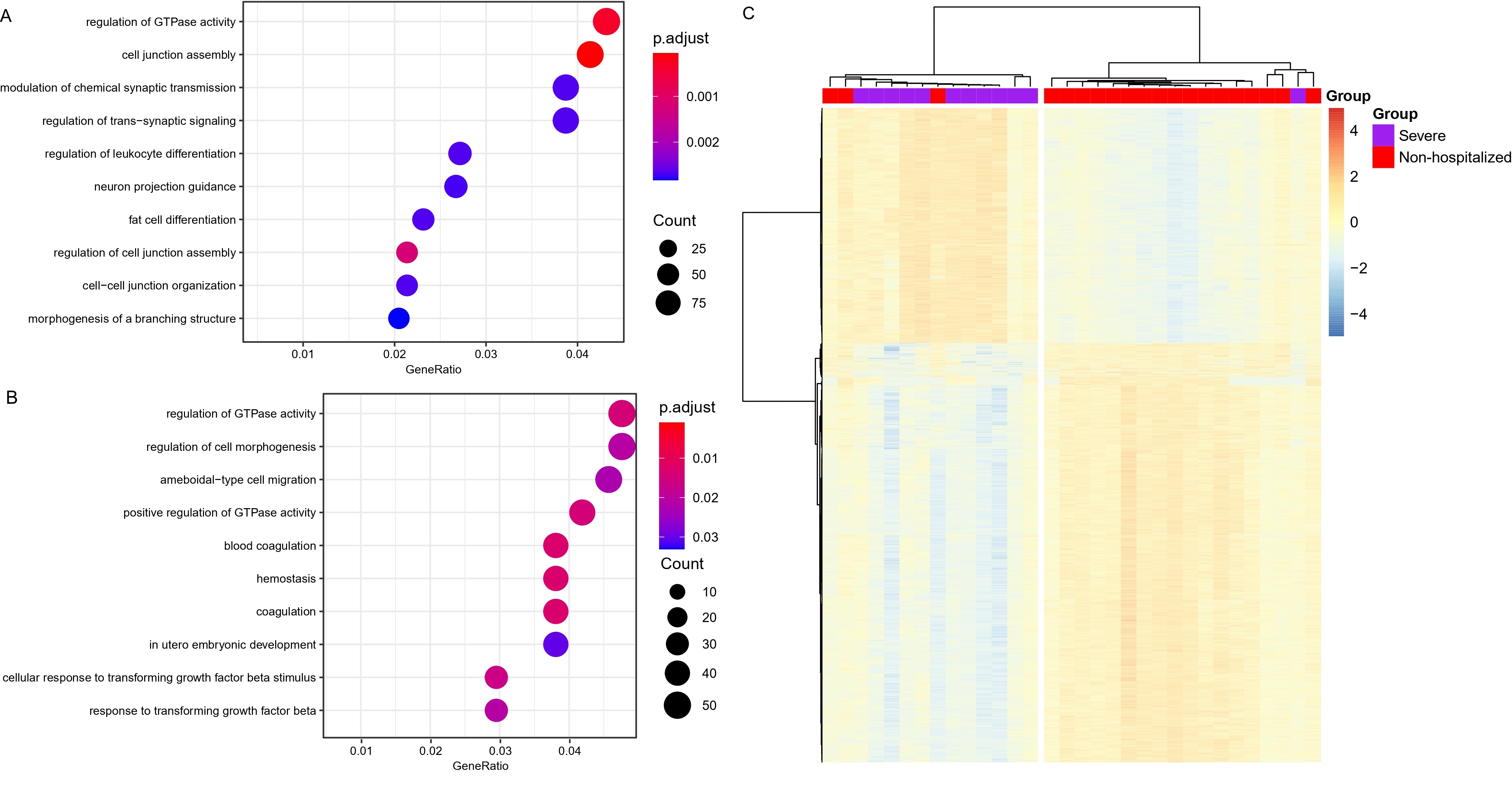

Supplement: Supplementary file 2 — Additional file 2: Fig. 1. Dotplots showing the top ten gene ontological (GO) biological processes related to the DMRs between A severe and healthy cohorts and B non-hospitalized and healthy cohorts; C heatmap of unsupervised clustering using differentially methylated regions between non-hospitalized and severe COVID-19 patients. [file 13148_2024_1645_MOESM2_ESM.jpg]
